# Does case-based blended-learning expedite the transfer of declarative knowledge to procedural knowledge in practice?

**DOI:** 10.1186/s12909-019-1884-4

**Published:** 2019-12-03

**Authors:** Bela Turk, Sebastian Ertl, Guoruey Wong, Patricia P. Wadowski, Henriette Löffler-Stastka

**Affiliations:** 1 Department of Psychoanalysis and Psychotherapy, Medical University of Vienna, Währinger Gürtel 18-20, 1090 Vienna, Austria; 2 Kennedy Krieger Institute, Johns Hopkins University, Baltimore, USA; 3 Faculté de Médecine, Université de Montréal, Montréal, Québec, Canada; 4 Department of Internal Medicine II, Division of Angiology, Medical University of Vienna, Vienna, Austria; 5 Teaching Center, Medical University of Vienna, Vienna, Austria

**Keywords:** Competence, Performance, Case-based learning, Case-based blended learning, Bloom’s taxonomy

## Abstract

**Background:**

Case-Based Learning (CBL) has seen widespread implementation in undergraduate education since the early 1920s. Ample data has shown CBL to be an enjoyable and motivational didactic tool, and effective in assisting the expansion of declarative and procedural knowledge in academia. Although a plethora of studies apply multiple choice questions (MCQs) in their investigation, few studies measure CBL or case-based blended learning (CBBL)-mediated changes in students’ procedural knowledge in practice or employ comparison or control groups in isolating causal relationships.

**Methods:**

Utilizing the flexibilities of an e-learning platform, a CBBL framework consisting of a) anonymized patient cases, b) case-related textbook material and online e-CBL modules, and c) simulated patient (SP) contact seminars, was developed and implemented in multiple medical fields for undergraduate medical education. Additionally, other fields saw a solo implementation of e-CBL in the same format. E- cases were constructed according to the criteria of Bloom’s taxonomy.

In this study, Objective Structured Clinical Examination (OSCE) results from 1886 medical students were analyzed in total, stratified into the following groups: medical students in 2013 (*n* = 619) before CBBL implementation, and after CBBL implementation in 2015 (*n* = 624) and 2016 (*n* = 643).

**Results:**

A significant improvement (adjusted *p* = .002) of the mean OSCE score by 1.02 points was seen between 2013 and 2015 (min = 0, max = 25).

**Conclusion:**

E-Case-Based Learning is an effective tool in improving performance outcomes and may provide a sustainable learning platform for many fields of medicine in future.

## Background

The concept of case-based learning (CBL) is a long-established didactic paradigm. In the last century, after having long been a mainstay in business and law school teaching [1], CBL has emerged in its current denomination as a central teaching tool in science and medical education, exhibiting key features advocated by educational researchers [2, 3]. This formalized teaching mode may have found its foundation in the Viennese pathologist Baron Carl von Rokitansky’s teachings on the correlation between pathological anatomy and disease presentation and course some 50 years ago [4]. The bridging of theory to practice is a common aim of CBL courses [4], as is the development and fostering of the transfer from declarative to procedural knowledge - training clinical reasoning. This transfer-learning in medical education has been highly advocated in a 2010 Lancet report [5]. Therein, Frenk J. and Chen L. et al. review the current global status of health education at the postsecondary level. E-Learning is seen as a very promising tool to revolutionize didactic approaches; however, the limitations, especially in developing countries, are also discussed [5].

Case-based discussion following a case presentation is a commonly used method of teaching in medicine. In pursuing a learning objective, the case may be openly discussed by students in a peer-teaching style, be guided by an instructor in a traditional didactic setting or, in an e-learning setting, take place in the form of conditional answer-specific feedback. Objectives of case-based discussion commonly relate to three specific cognitive aspects in students: the students’ own knowledge base, case specific details and principles of medicine [6]. Principles of medicine is a broad historic term and relates to a plethora of systems in patient treatment, described by Irby et al. as “the evidence based approach to patients within an ethical framework” [7]. While principles of medicine certainly do constitute declarative knowledge within the students’ “knowledge base”, they also encompass knowledge relating to ethical, optimal, and evidence-based approaches to the diagnosis and management of patients [8]. Based on this, as well as on the criteria of Bloom’s taxonomy [9], case structures are elaborated to depict clinical situations requiring higher order thinking skills [10–13].

Information theory (IT) has three major cornerstones: the activation of prior knowledge, the specificity of encoding, and the elaboration of knowledge [8]. Encoding specificity finds a solid base in translational neuroscience of memory formation in fear states [10]. Further, it refers to the fact that the more closely a situation where an item is learned resembles conditions in which it will be applied, the more likely encoding or “transfer of learning” will occur. Encoding specificity may be seen as the “active ingredient” and proves to be a salient feature of contextual learning theory in CBL.

### Case-based discussion in e-CBL

As we advance deeper into the twenty-first century, the new generations of students frequenting universities and medical schools are more and more composed of what Prensky first termed as “digital natives” in 2001 [14]: having known technology all their lives, these are “native speakers of the digital language of computers, video games, and the internet”, as he described them. These generations profess themselves as being more enthusiastic about (and dependent upon) technology and computers in their daily lives, and so not surprisingly, use it more heavily than previous generations do [15]. There is evidence to suggest that this has had repercussions on learning approaches: for example, in their 2018 study, Backhaus et al. [16] found that “digital natives” in their medical student study population performed significantly worse when learning under the “traditional” lecture format (standard in many medical schools) than when technology and e-learning resources were integrated.

Electronic CBL requires the student to self-engage in the case-based discussion. Training autonomous learning skills is desirable in fostering a commitment to lifelong learning. However, one major challenge of e-CBL is the requirement of a base of declarative knowledge (composed of both a knowledge base and principles of medicine) before engaging in e-CBL. While some benefit may be derived from engaging in e-CBL without prior fundamental declarative knowledge, achieving learning objectives in e-CBL with a solid knowledge foundation may improve case completion time, reinforce previously learned principles and declarative knowledge, and improve motivation. We also hypothesize that the learning benefit of e-CBL is optimal in a systematic, progressive, and multimodal learning framework: 1) Learning using written resources (i.e. textbook material) to generate and complete a knowledge base, followed by 2) e-CBL cases from real patients prior to 3) contact with real or simulated patients in order to apply newly clinical reasoning and decision-making skills.

### Does CBL improve performance measures?

CBL is considered to be a participatory teaching-learning method that facilitates active and reflective learning in students to develop critical thinking and effective problem-solving skills [17, 18]. More concretely, Beech and Domer [19] showed an increased mastery of physiology concepts, demonstrated by a pre- vs. post-intervention test. CBL dramatically improved exam scores in pediatric dentistry students, as those having been exposed to CBL outperformed their counterparts by nearly 20% [20]. Jamkar et al. [21] showed increased declarative knowledge scores in 6 groups of students (8–10 per group) in comparison to a control group of 55 students. A similar observation was also reported by Dietrich et al. [22] in 12 third year obstetrics/gynecology residents. Damjanov et al. [23] compared United States Medical Licensing Exam (USMLE) Step 1 exam results of three different year groups, showing significantly higher CBL-specific scores after a CBL-oriented curricular reform.

Furthermore, procedural knowledge, as measured by students’ competence in applying clinical reasoning skills, has also been shown to improve [24]. In fact, Deng [25] showed that CBL was associated with better diagnostic accuracy and treatment plans of resident doctors in training on a written examination.

Thistlethwaite et al. [4] however, note an important limitation of non-cohort studies without control groups in general, indicating that other teaching and learning methods in addition to CBL might also play a role in the observed improvements, and it would therefore be impossible to conclude that CBL alone was the causal factor.

While CBL has been demonstrated to have beneficial effects as a didactic method, there is relatively little data comparing CBL to other teaching methods with performance measures (i.e. OSCE scores) other than written exam test scores. We surmise that CBL may allow for greater identification with the role of the physician due to the structured approach in “working up” a clinical case.

### Student performance assessment methods and learning frameworks

In most of the aforementioned studies, knowledge and skills assessments are performed largely through the use of multiple-choice questions (MCQ) [4]. However, this surrogate parameter only allows reproduction of procedural knowledge in theory and not in practice. Use of these skills in a doctor- or student-patient communication training environment may be assessed using the OSCE.

Schwartz et al. [26] compared the efficacy of simulated patient (SP) training which employed electronic mannequins to CBL in medical students. Although no significant difference in performance was described, performance parameters were measured in OSCE scores, thereby evaluating procedural knowledge in practice.

Hull et al. [27] compared OSCE results between two groups, differing only in order of course completion: one was given bedside teaching (BT) and then CBL while the other saw CBL followed by BT. Interestingly, in this study, two OSCE examinations were performed, one after the first method, then again after completion of the other. Although after the first method, BT saw higher OSCE scores than CBL (*p* = .692), after completion of both interventions, the latter (CBL + BT) saw significantly higher scores (*p* = .038) than the former (BT + CBL).

In accordance with the conclusion reached by Hull et al. that CBL is useful and effective before BT or other forms of patient- or simulated patient-contact, we took further steps and implemented an e-CBL element in the curriculum, in order to highlight the importance of a progressive structure within a case-based blended learning (CBBL) framework. Now, it should be noted that there is no universally accepted definition of blended learning and what exactly it constitutes; indeed, it has been said that its different definitions are nearly as numerous as instances of its implementation [28]. However, here in the context of this study, the approach we have adopted can at least be said to adhere to its most classical definition: the integration of different learning approaches and technologies, heretofore generally achieved by combining traditional face-to-face instruction with online/computer-based activities [29, 30].

Several formulations of blended learning frameworks exist and have been tried elsewhere, each with unique strengths and weaknesses. Flipped classrooms, for example, in which teachers create online or computer-based instructional content to be viewed by students independently so that class time is (in theory) freed for more engaging (collaborative) activities [31], has been suggested as potentially an excellent means of teaching procedural knowledge [32]; however, the absence of an instructor while viewing the content presents challenges: students not being able to pose questions potentially critical to one’s mastery of the material, instructors being unable to monitor comprehension through formative assessments, etc. [33]. Massive online open courses (MOOCs), another novelty in educational models arising within the last decade, in which essentially anyone with access to an internet connection and a computer can access learning material and interact with other students [34], have been hailed for the flexibility they offer to students as well as their potential to make education accessible to a far larger audience [35]; unfortunately, low instructional quality and retention rates are pressing concerns [36, 37].

In a medical training context, face-to-face, in-person contact is an important component of clinical work, in our belief; which is why in our particular case, as we shall see, our blended learning approach integrated written textbook content, electronic material, and supervised clinical contact with simulated patients to achieve our aims. Overall, the goal of our e-CBL reform was to create a framework (compare with [38]) in which medical students may better acquire practical, applicable procedural knowledge and clinical reasoning skills, measurable through OSCE scores and testing.

## Methods

### Developing a framework for optimal e-CBL use: case-based blended learning (CBBL)

Our case-based blended learning (CBBL) approach consisted of a progressive three tier approach described in detail previously in Turk et al. [12], in which we had postulated that the transfer of declarative knowledge to procedural knowledge in theory, and then of procedural knowledge to procedural skills in practice, requires a three step multimodal approach. To summarize briefly, we developed, adapted, published, implemented, and evaluated our material for each of these three tiers, now described below.

A blended-learning (multimodal) approach using 1) textbooks, 2) interactive e-CBL cases transferred and created via the General Hospital electronic health records system [12, 13, 39, 40], and 3) simulated patient contact was designed and implemented university-wide for several subjects taught to medical students, starting with psychiatry in 2014 [12]. In total, our e-CBL framework and platform eventually saw adaptation and publication of relevant e-CBL cases for over 16 fields/sub-specialties in pre- and post-graduate medical education in total, including (but not limited to) psychiatry, pediatrics, neurology, infectious diseases, dermatology, microbiology, orthopedics, traumatology, internal medicine, surgery, and clinical genetics.

### Textbook/written resources

Case-relevant declarative knowledge was compiled in a textbook for each subject in which reforms were implemented [41]. This material was compiled in a case-based manner, created to work synergistically with as well as prior to the e-learning cases, thus serving as a declarative knowledge base. Each textbook chapter included sections describing: Anatomy and Physiology, Pathophysiology and Disease Process, and Epidemiology and Genetics. The goal of our case-based textbook was to create a relevant foundation of declarative knowledge, allowing its (future) transfer to procedural knowledge.

Each chapter section “Pathophysiology and Disease Process” was designed to overlap and be integrated with its corresponding presentation and physical examination in the e-CBL case. This crucial synergy aimed to foster a close link in creating the intimate connection between morphology and clinical presentation.

### Creation of E-CBL cases

E-CBL cases for each subject in which reforms were implemented were created using anonymized patient data from the Vienna General Hospital [12]. A novel plug-in for the existing electronic health records (ERH) system was developed, allowing automatic import and anonymization of real patient data from cases identified by attending physicians. Patient data was first transferred via the ERH into an anonymized case (for data protection purposes) on a separate platform. Following the initial transfer, the data was then automatically imported into the e-learning case template on an online administrator platform (Moodle). Two-stage validation was key to this step. Firstly, content creators (i.e. medical educators, physicians, etc.) were able to assess, edit and complete the cases for use in a teaching setting on Moodle, once the administrator gave them access privileges. Secondly, the completed cases had to be unlocked individually by the administrator and the content creator, before being placed in an online course available to students.

The e-CBL case templates were structured to enable data transfer to relevant sub-sections, thus greatly simplifying the editing and review process. Additionally, the template was optimized for case presentation following common case-presentation structures in medical education and was designed to complement the textbook chapters.

Each e-learning chapter included sections with relevant information on: Presentation and Communication, Physical Examination and Diagnostic Techniques, and Therapy and Prophylaxis.

Once case data was reviewed, content creators designed a virtual case-based discussion, in the form of multiple-choice questionnaires (MCQs). These MCQs allowed for multiple answers, with specific feedback for each correct or incorrect response, as well as follow-up questions. Their focus was specific to salient points in each chapter section (presentation and communication, physical examination and diagnostic techniques, therapy and prophylaxis).

MCQs focused either on clinical reasoning, requiring students to consider differential diagnoses, or on ordering the correct diagnostic test following a working hypothesis. To give a representative illustration of the types of questions/content developed and adapted for each subject, a model example in cardiology might be: 1) “Which of the following diagnostic tests should you initially order to assess the possibility of or rule out a myocardial infarction [for first time, sudden onset, level 10/10 VAS, crushing substernal chest pain] without dyspnea?

A) 12 lead EKG [Correct]
B) Trans-thoracic echocardiography
C) Chest X-ray
D) Computed tomography”

In creating such case-based discussions, feedback was given for each possible answer (if selected), written by the content creators, i.e. why one choice is correct and not another, or if both choices seem correct, why one is prioritized over the other. This feedback allowed the students to confirm, practice, or retroactively develop procedural knowledge.

For the model question above, the feedback reviewed case-specific data in the context of declarative knowledge and/or expanded on possible alternative outcomes:

Initial testing must be performed in a timely manner, starting with the most relevant non-invasive procedure.

- [Specific feedback for A)]: A 12 lead EKG should be obtained within 10 min of presentation for all patients with chest pain. Morphological changes such as ST-segment elevations > 1 mm in > 2 anatomically contiguous leads (STEMI) or a novel left bundle branch block; non-ST-elevation MI (NSTEMI) or unstable angina: ST-segment depression or T-wave inversion. Morphological changes in conjunction with notable heart parameters in blood testing warrant immediate emergency management.
- [Specific feedback for B)]: Trans-thoracic echocardiography (TTE) is not considered in initial testing for myocardial infarction, as performing and reading TTEs is not the most efficient and sensitive method. Pathological features such as wall motion abnormalities, valve disease and congenital heart disease may be assessed as well as prognostic data garnered from color-flow Doppler transthoracic echocardiography. Sensitivity is highly dependent on the ability to appropriately image the apex and sensitivity may be as high as 90%. However, timely diagnosis is key in determining the emergency nature of the illness.
- [Specific feedback for C)]: A chest X-ray should be routinely performed on all patients presenting with chest pain. However, the sensitivity and specificity of findings indicative of myocardial infarction are low.
- [Specific feedback for D)]: Computed tomography is not considered in initial work-up of chest pain, due to the time needed to complete- and the ionizing radiation caused by the procedure. Specificity and sensitivity for the diagnosis of MI using CT angiography is above 95%, allowing the visualization of ventricular aneurysms and intra-coronary thrombi.

### Simulated patient contact

Students were required to successfully complete the e-learning course by correctly answering all questions before taking part in the seminar. The SP seminar has the aim of applying and transforming procedural knowledge into procedural skills, while also allowing students to experience, document, and reflect on difficulties that arise from newly encountered dimensions of face-to-face communication with mentally ill patients [42]. Here the student is required to observe, give feedback, and perform a complete psychiatric consultation, including conducting a mental status examination and taking a psychiatric history, creating a clinical or diagnostic hypothesis, deciding on further case management, and finally suggesting relevant therapy options, thus integrating all previously acquired skills.

The SP employs the use of professional actors who have received training in embodying patients, requiring students to apply both the declarative knowledge and clinical reasoning skills learned in the textbook and e-learning cases. Actors learn their “roles” using prepared and anonymized patient case data. After the course, students are assessed by educators, peers, and themselves [43], with communication portfolios documenting their simulated consultations.

### Data collection

Anonymized OSCE scores of all medical students at the Medical University of Vienna who partook in the clinical psychiatry examination “Physician communication skills” [Ärztliche Gesprächsführung, ÄGF-C] of the 4th year OSCE in the years 2013, 2015, and 2016 were retrospectively retrieved by H.L-S. from university student records’ electronic databases and analyzed. The data protection committee of the Medical University of Vienna, independent of anyone involved in this study, first anonymized all data before allowing access, and also approved this study.

Evaluated data sets included the OSCE scores from the respective cohorts of 4th year medical students in 2013 (before any implementation of the CBBL framework, thus considered “pre-intervention”), 2015 (considered thus “post-intervention”), and 2016 (also considered as being “post-intervention”, included for verification purposes, so that if any significant improvement were to be observed between 2013 and 2015, a similar improvement observed as well in 2016 vs. 2013 would be an indicator that such improvements were more likely to be a persistent and reproducible effect of CBBL implementation, rather than merely a coincidence).

### Statistical methods

Non-linear regression analysis was performed, and datasets were tested for normality and homogeneity of variance. The Shapiro-Wilk test by Royston [44] and assessment of kurtosis and skewness using the D’Agostino-Pearson omnibus test [45] were carried out in order to determine the normality of distribution of each cohort year’s OSCE scores. A Brown-Forsythe test [46] was carried out in order to test homogeneity variance between groups.

Data pairing was considered, as test takers for 2013, 2015, and 2016 may be seen as being matched by level of progression of medical education (all test takers being in the 4th year (out of 6) of medical school in these respective years). However, considering the presence and possible effects of a host of unknown variables such as age, sex, ethnic group, and the number of exam attempts, etc., we felt that unpaired testing was to be favoured. Thus, comparison between cohort year datasets was performed using nonparametric, non-paired t-tests (Mann-Whitney [47]). Although the consensus is that non-parametric tests commonly have less power [48], as will be seen later, they were employed due to our non-normal data distribution.

Then, comparison of the medians of the three groups was performed through use of the Kruskal-Wallis H test [49], assuming non-parametric data distribution. Also due to our non-normal data distributions, Chi value generation for the Kruskal-Wallis test was performed using Murphy & Myors’ [50] transformation into an F value prior to Laken’s adapted Cohen’s formula [51], providing an eta value instead of a more common Cohen’s d. Following that, Dunn’s multiple comparison tests [52] were used post hoc to compare groups within a non-parametric ANOVA. For effect size between groups, Rosenthal & DiMatteo’s [53] effect size calculation was performed.

In this study, statistical significance was assumed by a *p*-value < 0.05.

## Results

### 2013 OSCE scores

OSCE results from the year 2013 and a total of *n* = 619 students are considered pre-intervention. Median score was 19 points (25% percentile: 16, 75% percentile: 23 points), the mean score was 18.76 points (standard deviation (SD) 4.43, standard error of the mean (SEM) .1792 points).

Normal distribution could not be assumed as shown by the Shapiro-Wilk test (W = 0.9516), *p* < .0001, and the D’Agostino-Pearson omnibus test K^2^ = 71 (*p* < .0001), Skewness = − 0.2013, Kurtosis − 0.8672. Negative skewness confirms the long left tail. Considering the negative kurtosis, a platykurtic distribution may not be assumed due to large outlier of highest frequency at 25 points.

### OSCE 2015 scores

OSCE results from the year 2015 from a total of *n* = 624 students are considered “post-intervention”. Median score was 20 points (25% percentile: 17, 75% percentile: 22 points), the mean score was 19.78 points (SD 3.175, SEM 0.1342 points).

Normal distribution could not be assumed, using the Shapiro-Wilk test (W = 0.9689), *p* < .001, and the D’Agostino-Pearson omnibus test K^2^ = 32.21 (*p* < .001), skewness = − 0.2886, kurtosis = − 0.6453.

### OSCE 2016 scores

OSCE results from the year 2016 from a total of *n* = 643 students are also considered as being “post-intervention”. Median score was 21 points (25% percentile: 19, 75% percentile: 23 points), the mean score was 20.29 points (SD 3.383, SEM 0.1342 points).

Normal distribution could not be assumed using the Shapiro-Wilk test (W = 0.9427), *p* < .001, and the D’Agostino-Pearson omnibus test K^2^ = 47.73 (*p* < .001), skewness = − 0.7329, kurtosis = 0.1402.

### Comparison of 2013 and 2015 OSCE scores

In comparing pre-intervention results from the 2013 OSCE to post-intervention 2015 OSCE results, a Mann-Whitney U test indicated that the median test scores were significantly higher in 2015 (median = 20 points) than in 2013 (median = 19 points), with U = 164,991, *p* = .002.

### Comparison of 2015 and 2016 OSCE results

A comparison of post-intervention OSCE scores between 2015 and 2016 employing a Mann-Whitney U test showed significantly higher mean scores in 2016 (median = 21 points) versus 2015 (median = 20 points), U = 174,420, *p* = .009.

### Comparison of 2013, 2015, and 2016 OSCE results (Fig. 1)

The Brown-Forsythe test performed to test homogeneity variance between groups was highly significant for a violation of an assumption of homogeneity (F = 49.77 (2, 1860), *p* < .0001). Given the non-normal data distributions and inhomogeneity of variance between groups, results of a one-way parametric ANOVA could not be reliably assessed, and thus was not performed.

**Fig. 1:**
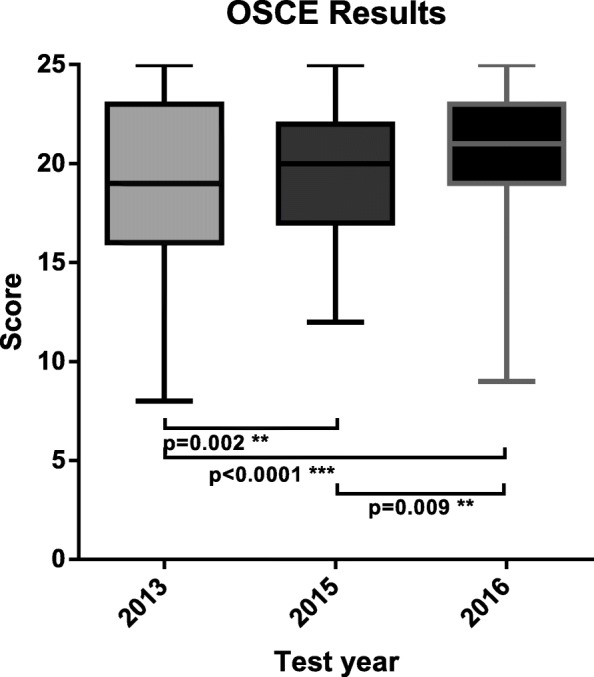
Boxplot comparison of medical students OSCE test scores from 2013 (*n* = 619), 2014 (*n* = 624) and 2015 (*n* = 643). Kruskal-Wallis one-way ANOVA showed significant differences between the non-parametric yet similarly distributed 2013, 2015 and 2016 OSCE test results

While not as powerful as a parametric one-way ANOVA, the non-parametric Kruskal-Wallis test compares the “sum of the ranks” [54]. It was chosen instead, due to the Kruskal-Wallis assumption that the distribution of each data set is of the same non-parametric “type” and differ from one another in their median. As skewness of the data sets was all left tailed, i.e. negative (2013 skewness: − 0.2013, 2015 skewness: − 0.2886, 2016 skewness: skewness = − 0.7329), we may cautiously accept inference in terms of dominance in these data distribution. Thus, an interpretation of significance may be considered equal to a comparison of the medians.

The Kruskal-Wallis H test showed a statistically significant difference between the OSCE scores from 2013, 2015 and 2016 (H (5.991, *n* = 619) = 40.45, *p* < .0001). Dunn’s post-hoc multiple comparisons test determined statistically significant means in 2013 vs. 2015 (H (14.023) with adjusted *p* = .002), in 2013 vs. 2016 (H (35.843) with adjusted *p* < .0001), and in 2015 vs. 2016 (H (10.578) with adjusted *p* = .009).

As data distribution violated the assumption of a normal distribution, effect size of the intervention could not be measured by Cohen’s formula alone, so instead Murphy & Myors’ transformation of the Kruskal-Wallis [50] chi^2^ into an F value was performed prior to application of Laken’s adapted Cohen’s [51] formula for ç^2^ for three groups, F(5.991), ç^2^ = 0.01. For effect size between groups, Rosenthal & DiMatteo’s effect size calculation [53] gave R(3,841), ç^2^ = 0.08.

## Discussion

CBBL was implemented at the Medical University of Vienna, following a planned curricular change first agreed upon in 2012 [Curriculum Novelle], which aimed to establish the clinical practical year in the final (6th) year of medical school. Initially tried in psychiatry teaching in order to better prepare students for the practical difficulties of handling certain patients, it was later expanded and applied to several more subject areas with clinical aspects in the hope of improving practical clinical reasoning and decision-making skills in these subjects as well. While similar e-CBL systems have seen some implementation and improvements have been shown in test-scores in paired groups with smaller sample sizes in other studies, the authors are not aware of similar large data sets in medical education (as presented in this study) that address variables influencing performance parameters in evaluating the use of e-CBL.

Analyzing and evaluating the impact of its introduction through effect on performance measures (such as test scores), however, is complicated by manifold variables in the education setting. One such difficulty in particular is in separating the individual effects of such intimately related variables from each other, such as learning material, teacher effects, classroom-specific factors, and test-specific aspects (test difficulty/preparation). This makes ascertaining the acquisition of knowledge due to our curricular reforms alone especially challenging [55]. Isolating the true impact of CBBL on our exam score data from other potential confounders is a difficult task, therefore, and so one should naturally be cautious when interpreting these results.

### Analysis of effect size and significance

Effect size η^2^ values of < 0.20 are generally considered small, ≈ 0.50 medium and > 0.80 large [56]. An η^2^ value of 1.0 in a medical education setting would require a student initially at the 10th percentile to improve to the middle third and a student in the 25th percentile to improve to the third quartile. While this would be especially impressive of any intervention in medical education, assessment of test scores as a performance measure is confounded by the “ceiling effect”.

The “ceiling effect” refers to top performing subjects receiving the highest or close to highest score on the measuring scale in the initial or pre-intervention measurement. The potential for improvement and/or response to intervention is thus limited, as they simply cannot attain a higher score following intervention.

The 2013 OSCE score data is not normally distributed, as the highest frequency of scores is the highest score at 25 points. The effect of this distribution on further analysis is a clear indication of the ceiling effect, greatly impinging on statistical calculations of effect size following intervention (e.g. 2013 vs 2015, η^2^ = 0.08).

For even a small effect size to be observed, the scores of students at the lower end would have to drastically increase in the 2015 or 2016 OSCE, and an overall “small effect size” (η^2^ = 0.20) would require an improvement from 2013’s mean of 18.76 points to a theoretical mean score of 24.22 points! Instead, the improvement of mean scores to 19.78 points in 2015 (*p* = 0.002) and 20.29 points in 2016 (*p* < 0.0001) was statistically significant, albeit with a very small associated effect size (η^2^ = 0.08).

Additionally, due to the non-parametric pre-intervention data set caused by the ceiling effect, statistical models used in data analysis had to be chosen rigorously, lowering the power of the analyses as opposed to optimal employment of Student’s t-tests or parametric ANOVAs.

### Test difficulty is a confounding variable

The OSCE in 2013, 2015 and 2016 saw the use of different questions and different examiners. Therefore, the data distribution in 2013 resulting in a statistical ceiling effect may be due to an easier test in 2013, followed by a more difficult test (and thereby a more Gaussian distribution with a highest frequency of scores in a more high/mid-range) in 2016. Ideally in the future, data on the difficulty of examination tasks or questions should be complied and assessed, in addition to the raw scores themselves, in order to account for the year-to-year variability of test difficulty. This might possibly be achieved by a student-rated evaluation of test difficulty; however, this itself may be confounded by other factors such as the students’ degree of preparedness for the exam, amongst others.

However, as the mean score still improved significantly (*p* = 0.002) by 1.02 points, despite a shift in data distribution from 2013 to 2015 as well as possibly a more challenging test, we may cautiously assume a gain of knowledge due to the curricular modification, although of course, one may argue that the opposite case is always possible (that the 2013 version of the exam was actually found to be more difficult compared to the later versions, also a possible explanation). The 2016 cohort did still see a significant improvement by 0.51 points, compared to 2015. The CBBL curriculum had not been altered in that year from 2015 and the Gaussian distribution of the 2016 scores may also indicate a similarly difficult test to the 2015 test in comparison to 2013. However, this 2016 improvement may very well also be influenced by a change in test difficulty compared to 2015. Also, the role of possible intrinsic differences between student cohorts (inter-cohort variability in terms of “competence”) in these results must not be neglected either.

### A critical perspective on E-CBL

In establishing an e-CBL program, one must see to careful assessment of performance outcomes. In this study, it should be noted that comparison of “pre-” and “post-intervention” OSCE scores was limited to one subject area (clinical psychiatry). Evaluation of performance outcomes should ideally be performed in the other fields of medicine where e-CBL has been implemented in order to validate these data. In future studies, more established and comprehensive exams, such as the summative integrative examination [German: *Summative Integrative Prüfung*] (SIP), may provide a more rigorous data set for evaluation.

From a practical standpoint, one major uncertainty about implementing e-learning is due to a distinct lack of data on its total cost [57]. While a net saving value is highly praised [58], other data suggest high development costs in relation to existing network infrastructure [59, 60].

Another critical aspect of e-CBL is the perceived time and workload involved in the design and content creation of e-cases [61]. One analysis of work-time spent for academic content creators saw a total of 12 h (7.3 academic, 3.3 technical and 1.4 administrative) hours spent per hour of student online activity [61]. A radical reduction of time investment is described following creation of the first e-case. Our workgroup confirms the time reduction once content creation becomes routine.

In our CBBL case-generation phase, time investment was not measured. The hurdle of a perceived large investment of time and other resources was overcome by frequent training and technical support from the CBBL task force, in addition to the support of residents and medical students in assisting content creation.

While distance teaching has been unequivocally shown to require a higher time-investment [62–64], a growing need for e-CBL development has equally been stressed in the literature [65].

Academic content creation is a cornerstone of university education. Rumble et al. stressed that pressuring academics to create e-learning resources might constitute a “hint of exploitation” and called for caution when working on course development [66]. Brogden and Howell [62, 67] agreed that the greatest obstacle to the development of e-learning and other such resources is the labor- and time-intensive demand on the content creators.

Alexander et al. [65], as well as McPherson & Nunes [68] suggest the need for an institution-wide e-learning plan regarding implementation and evaluation, interdisciplinary collaboration, and appropriate levels of support. When the current e-CBL format [12] was initially presented to six departments at the Medical University of Vienna, interdisciplinary communication and collaboration was facilitated by bi-monthly meetings with academic representatives from each field. In our opinion, the continuous support and direction offered from such regular meetings is essential to implementation and maintenance of a medical program that best favours medical students’ acquisition of clinical reasoning and decision-making skills.

## Conclusion

A case-based blended learning (CBBL) framework might be employed by medical programs in order to foster interdisciplinary learning, first by developing competencies for clinical reasoning and decision-making in each discipline and then integrating them together. In addition, e-learning as a general premise is already part of the teaching philosophy of many schools, and thanks to the controlled and more relaxed learning process it offers, has even been suggested as a possible means of preventing burnout and disillusionment [69].

Implementation of e-cases created on the basis of a large hospital’s electronic health records system can facilitate learning and, in our opinion, should be transferred to an ongoing continuous learning platform to assist and prepare bedside teaching. Progression and advancement from simpler cases through to more complicated ones can assist documentation and testing of a student’s learning progress [70, 71], as well as be used for curricular development and harmonization with corresponding medical curricula in other countries. This could offer the additional benefit to students, from an international perspective, of greater mobility to study and/or work elsewhere much more easily, as well as reduce barriers to obtaining licensing and certification to practice medicine in foreign countries.

## Data Availability

The datasets generated and analyzed during the current study are not publicly available due to reasons of confidentiality according to the data protection committee of the Medical University of Vienna but are available from the corresponding author upon reasonable request.

## Abbreviations

ÄGF-C: “Physician communication skills” [“Ärztliche Gesprächsführung-C“]
BT: Bedside teaching
CBBL: Case based blended learning
CBL: Case Based Learning
e-CBL: Online Case Based Learning
IT: Information theory
MCQs: Multiple choice questions
OSCE: Objective Structured Clinical Examination
SP: Simulated patient
USMLE: United States Medical Licensing Exam
